# The courtship choreography of homologous chromosomes: timing and mechanisms of DSB-independent pairing

**DOI:** 10.3389/fcell.2023.1191156

**Published:** 2023-06-12

**Authors:** Mireia Solé, Álvaro Pascual, Ester Anton, Joan Blanco, Zaida Sarrate

**Affiliations:** Departament de Biologia Cel·lular, Genetics of Male Fertility Group, Unitat de Biologia Cel·lular, Fisiologia i Immunologia, Universitat Autònoma de Barcelona, Barcelona, Spain

**Keywords:** homologous chromosomes, homologous pairing, chromosome dynamics, meiosis, cell division

## Abstract

Meiosis involves deep changes in the spatial organisation and interactions of chromosomes enabling the two primary functions of this process: increasing genetic diversity and reducing ploidy level. These two functions are ensured by crucial events such as homologous chromosomal pairing, synapsis, recombination and segregation. In most sexually reproducing eukaryotes, homologous chromosome pairing depends on a set of mechanisms, some of them associated with the repair of DNA double-strand breaks (DSBs) induced at the onset of prophase I, and others that operate before DSBs formation. In this article, we will review various strategies utilised by model organisms for DSB-independent pairing. Specifically, we will focus on mechanisms such as chromosome clustering, nuclear and chromosome movements, as well as the involvement of specific proteins, non-coding RNA, and DNA sequences.

## Introduction

Meiosis is a process aimed at producing haploid gametes from diploid germ cells. With this purpose, a single round of DNA replication is followed by two consecutive chromosome segregations. Meiosis increases genetic variation via two important mechanisms, namely, independent assortment of homologous chromosomes and genetic recombination. To this end, it is required that, in meiosis I, homologous chromosomes come close together in a process called pairing, synapse via synaptonemal complex (SC) formation (reviewed in Page and Hawley, 2004), recombine (reviewed in Hunter, 2015) and segregate randomly. Although these four processes are conceptually distinct, they are all closely related and take place in a sequential way.

It is widely accepted that the generation of DSBs by the topoisomerase-like transesterase protein Spo11 and the subsequent action of the DNA repair machinery (reviewed by Keeney, 2008; Baudat et al., 2013) induce the physical recognition among homologous DNA sequences. Once DSBs have been formed, the ends are resected to generate 3’ single-strand tails, which are loaded with RecA-like proteins, Rad51 and Dmc1. Proteins and DNA form a filament (via a nascent D-loop) able to identify and interact with their corresponding homologous double-strand DNA, that eventually cause the approach and coalignment, at a distance of approximately 400 nm, of specific regions of homologous chromosomes—the PAIRING (reviewed in Zickler, 2006). It has been suggested that only one of the two generated ends would create this “homology searching tentacle” of DNA and nucleoproteins (Kim et al., 2010). The alignment of the entirety of the homologous chromosomes requires the assembly of SC—the SYNAPSIS (reviewed in Page and Hawley, 2004). Subsequently, the process of RECOMBINATION will move forward through different strand isomerisations leading to crossover and non-crossover products (reviewed in Hunter, 2015; Gray and Cohen, 2016).

Accordingly, in some species the formation and repair of DSBs play an essential role in the processes of pairing and synapsis. In support of this hypothesis, it has been observed in *spo11* mutants a relationship between alterations in the number of DSBs and anomalies in the formation and functionality of the SC (Baudat et al., 2000; Romanienko and Camerini-Otero, 2000; Tesse et al., 2003; Kauppi et al., 2013; Rockmill et al., 2013). Moreover, exogenous DSBs induction partially restores the meiotic defects observed in some of these mutants (Thorne and Byers, 1993; Dernburg et al., 1998; Storlazzi et al., 2003; Tessé et al., 2003).

In contrast, in certain model organisms such as *Drosophila* or *Caenorhabditis*, the involvement of DSBs in the pairing process seems to be dispensable. Moreover, regardless the participation of DSBs, several aspects of the pairing mechanism indicate the existence of alternative pathways that play a role in facilitating the recognition and alignment of homologous chromosomes. For instance, it should be noted that each DSB generates approximately 1 kb of ssDNA that needs to identify and localise its homologous chromatid. A homologous sequence search should be achieved within a short period of time and then held together. This action is not that simple if homologous chromosomes are not previously sharing the same territory. Furthermore, chromosomes contain repetitive sequences, and thus, potential interactions between these pseudo-homologous regions must be avoided or eliminated during the homology search process.

In this article, we review the strategies described in different model organisms that promote homologous pairing throughout mechanisms not related to DSBs formation. It is important to note that the term “pairing” will be used to describe the approximation, association and recognition of homologous chromosomes before the onset of synapsis.

### Saccharomyces cerevisiae

**FIGURE 1:**
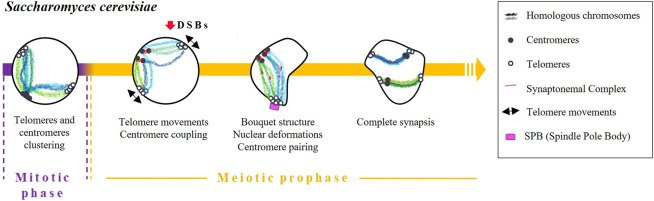
Timing and mechanisms of DSB-independent homologous pairing in *Saccharomyces cerevisiae*.

The initial stages of homologous pairing in budding yeast are determined by telomere clustering and centromere coupling (Figure 1). In vegetative (mitotic) cells, telomeres are located in a few clusters at the periphery of the nucleus. After the induction of meiosis, telomeres disperse over the nuclear periphery and cluster at the spindle pole body (SPB) (Trelles-Sticken et al., 1999). Meiocytes arrested in premeiotic S-phase have only a few peripheral telomere clusters, suggesting that the resolution of peripheral vegetative telomere clusters occurs at the end of or shortly after premeiotic S-phase (Trelles-Sticken et al., 1999; Trelles-Sticken et al. 2000; Trelles-Sticken et al. 2005). Then, during prophase I, telomeres are distributed in a rim-like pattern (Trelles-Sticken et al., 1999; Trelles-Sticken et al., 2000) and move rapidly (Trelles-Sticken et al., 2005) to create miniclusters that eventually assemble into the large cluster that characterises the bouquet stage (Trelles-Sticken et al., 2005). Once the bouquet is formed, telomeres continue to move rapidly, and the nucleus undergoes oscillating deformations (Trelles-Sticken et al., 2005; Koszul et al., 2008).

Although the molecular mechanisms regulating telomere attachment and clustering during meiosis are not well understood, the presence of the meiotic telomere specific adaptor protein Ndj1/Tam1 appears to be essential for this process (Chua and Roeder, 1997; Conrad et al., 1997). Additionally, it has been observed that telomeres experience an actin-dependent constraint on their mobility during the bouquet stage of meiosis. Cohesin is required to exit the actin polymerisation-dependent telomere clustering and link the SPB to the telomere clustering (Trelles-Sticken et al., 2005).

As soon as pre-meiotic chromosome replication is finished, centromeres undergo homologous and non-homologous pairwise associations, a phenomenon known as “centromere coupling” (Tsubouchi and Roeder, 2005; Obeso and Dawson, 2010). Remarkably, the formation of DSBs and the resulting signalling pathways are not essential for this phenomenon as demonstrated by observation that coupling occurs in mutants lacking the Spo11 protein (Tsubouchi and Roeder, 2005; Obeso and Dawson, 2010). Conversely, the absence of the SC component Zip1 resulted in undetectable centromere coupling, demonstrating the crucial function of this protein in the process (Obeso and Dawson, 2010). Cohesin, on the other hand, is believed to be also required for centromere coupling due to its influence on Zip1 localization rather than its direct participation in the coupling process (Chuong and Dawson, 2010).

Subsequently, as synapsis between homologous chromosomes begins, centromeres seem to transition from centromere coupling to centromere pairing, which involves the specific association of homologous centromeres (Tsubouchi and Roeder, 2005; Storlazzi et al., 2010; Lake et al., 2015).

The cause of centromere coupling is still not fully understood, but some studies have proposed that chromosomes have a partner selection preference dependent on their length (Lefrançois et al., 2016) that may contribute to the effectiveness of homologous pairing in the later stages of meiosis. Besides, it has been suggested that centromere pairing can serve as an alternative mechanism to link achiasmate homologous chromosomes (Dawson et al., 1986). In fact, observations have been made of achiasmatic chromosomes pairing specifically at their centromeres, providing evidence for this alternative pairing mechanism (Kemp et al., 2004; Gladstone et al., 2009; Newnham et al., 2010).

### Schizosaccharomyces pombe

**FIGURE 2:**
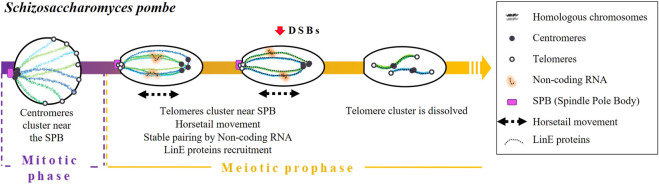
Timing and mechanisms of DSB-independent homologous pairing in *Schizosaccharomyces pombe*.

Homologous pairing in fission yeast is initiated during the mitotic replication phase and achieved by a combination of different mechanisms acting in an orchestrated way: centromeres and telomeres clustering, nuclear movements, as well as the accumulation of non-coding RNA and the presence of specific cohesins (Chikashige et al., 1994; Ding et al., 1998; Ding et al., 2012; Elkouby et al., 2016; Rubin et al., 2020) (Figure 2). It is worth emphasizing that pairing and synapsis take place normally in *rec12* mutants (*spo11* homolog) (Ding et al., 2012). This observation strongly suggests that both processes are independent of DSBs.

During the mitotic replication phase, the centromeres of *S. pombe* are grouped in association with the SPB (Funabiki et al., 1993; Chikashige et al., 1997). Once meiosis begins, immediately after karyogamy, centromeres detach from SPB and telomeres slide through the nuclear envelope and cluster forming a *bouquet* structure (Chikashige et al., 1994; reviewed in Hiraoka and Dernburg, 2009). It has been established that the loss of telomere-SPB clustering by mutations of telomere binding proteins, such as Taz1 or Rap1 (two proteins involved in telomere maintenance) or by mutations of the Kms1 membrane-bound SPB component, reduces recombination frequencies (Shimanuki et al., 1997; Cooper et al., 1998; Nimmo et al., 1998; Chikashige and Hiraoka, 2001; Kanoh and Ishikawa, 2001).

Then, the nucleus elongates and undergoes a movement called “horsetail”. This movement consists of going forward and backward of the cell (Chikashige et al., 1994; Ding et al., 1998) and will eventually allow the achievement of pairing and recombination (Ding et al., 2004). In dynein-disrupted meiotic cells, there is a lack of nuclear movements that end up in paring anomalies (Ding et al., 2004) and low recombination levels (Yamamoto et al., 1999).

In the end, horsetail movements result in stable pairing through the participation of the *sme2* locus. This gene encodes a non-coding RNA required for homologous recognition (Watanabe and Yamamoto, 1994), which is retained at the *sme2* locus by a set of specific proteins (sme2 RNA-associated protein; Smp) (Ding et al., 2016a). It has been proposed that the accumulation of non-coding RNA acts as a recognition marker of DNA sequence homology (Ding et al., 2016b). Indeed, other loci containing genes that encode for long non-coding RNAs have been described as essential for homologous chromosome recognition in different situations. For instance: the X-Inactivation centre encoding the long non-coding RNAs *Xist* in X-chromosome inactivation in mammalian females (Siniscalchi et al., 2022).

Horsetail movements are also associated with the establishment of a SC-like structure between homologous chromosomes formed by the linear elements (LinEs) proteins (Olson et al., 1978; Hirata and Tanaka, 1982; Bähler et al., 1993; Ding et al., 2012), which are evolutionarily related to the axial/lateral elements of the SC. Ellermeier et al. (2005) proposed that the linear element component Rec10 is recruited, which in turn activates Rec12 to perform DNA breaks (Ellermeier et al., 2005). Core LinE proteins (Rec10, Rec25, Rec27, and Mug20) are present only during the horsetail stage except the LinE-binding protein Hop1, which does not disappear even after meiosis I chromosome segregation (Ding et al., 2012). Once movements are finished, telomere clustering dissolves, and homologous chromosomes remain paired (Chikashige et al., 1994; Yamamoto et al., 1999; 2001; Ding et al., 2004).

Finally, Ding et al. (2016a) demonstrated that cohesins also contribute to homologous pairing since it was significantly impaired in *rec8* and *pds5* mutants.

### Drosophila melanogaster

A distinctive feature of *D. melanogaster* is that homologous chromosomes are paired in somatic cells. This feature called “somatic pairing” (Metz, 1916) is frequently observed in Dipterans (Metz, 1916; McKee, 2004; Joyce et al., 2016; King et al., 2019). It has been proposed that somatic pairing initiates at discrete sites (“the button model”) along the length of each chromosome (Funabiki et al., 1993; Rowley et al., 2019; Viets et al., 2019). Interestingly, some topologically associated domains (TADs) seem to conduct homologous associations, acting as high affinity pairing sites (Viets et al., 2019). In fact, “buttons” also drive pairing with their homologous sequences even when placed at different positions in the genome (Viets et al., 2019).

**FIGURE 3:**
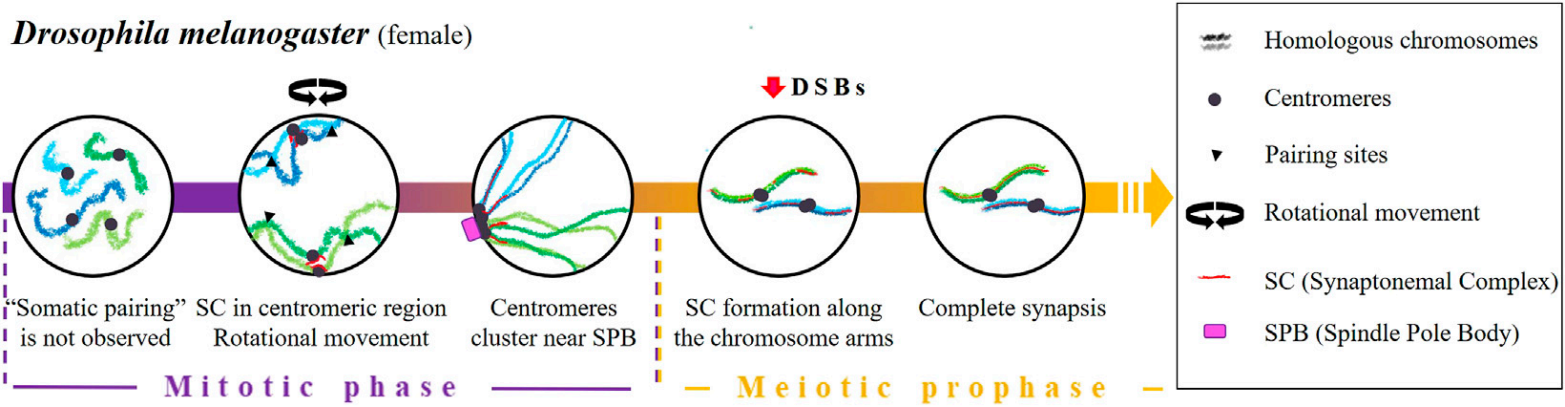
Timing and mechanisms of meiotic homologous pairing in *Drosophila melanogaster*.

Concerning meiotic cells, homologous pairing was thought to be an extension of a supposed pre-existing pairing in premeiotic germ cells (Stevens, 1908; Metz, 1926; Brown and Stack, 1968). However, it was observed that, during the first stages of oogenesis, homologous chromosomes remain unpaired in primordial germ cells [except for the specific repetitive sequences in the ribosomal DNA (rDNA)] (Christophorou et al., 2013; Joyce et al., 2013). Pairing is progressively re-established during the mitotic phase, before the onset of meiosis and the formation of DSBs (Vazquez et al., 2002; McKee et al., 2012), through the bundling of centromeres into clusters (Takeo et al., 2011; Christophorou et al., 2013; Joyce et al., 2013) near the SPB (Zou et al., 2008) and the aggregation of pairing sites (McKee and Karpen, 1990; McKee et al., 1992) (Figure 3).

The mechanisms that lead to centromere clustering before the onset of meiosis are poorly understood. In female *D. melanogaster*, two key factors have been proposed: the presence of SC elements in the centromeric region (Christophorou et al., 2013) and the rotation of the nucleus (Christophorou et al., 2015). Concerning the role of SC elements, two proteins C (3)G and Corona (CONA), which are associated with the transverse filaments and central element of the SC, respectively (Page and Hawley, 2004; Anderson et al., 2005; Page et al., 2008) show a direct relationship between their levels of accumulation in the centromeres of mitotic germ cells and centromere clustering. Homologous pairing is reduced by 30% in *C (3)G* and *Cona* female mutants that also display defective clustering (Christophorou et al., 2013). On the other hand, Christophorou et al., 2015 observed that in female *D. melanogaster,* the rotational movement of the nucleus during mitotic cycles contributes to homologous pairing. In their work, they demonstrate that microtubules, centrosomes, the motor proteins dyneins as well as the Sun and Kash domain transmembrane proteins (which play critical roles in establishing the connection between the nuclear envelope and the cytoskeleton) are required for centromere motion, pairing, clustering and homologous chromosome synapsis.

It is important to mention that the homologous recombination program promoted by DSBs starts shortly after the initiation of SC formation along the chromosome arms (Liu et al., 2002; Mehrotra and McKim, 2006; Lake et al., 2011) and it is not needed for the centromeric aggregation (Takeo et al., 2011). In *Mei-W68* mutants (lacking the enzyme responsible for catalysing DSB formation) and *Mei-P22* mutants (lacking the enzyme that facilitate DSB formation by MEI-W68), which are characterized by the absence of meiotic recombination, a normal SC formation is observed (McKim et al., 1998). However, in the absence of the SC proteins C (3)G and C (2)M, the number of DSBs in oocytes is significantly reduced (Mehrotra and McKim, 2006), suggesting that SC proteins are required for DSB formation.

In male *D. melanogaster*, there is no evidence of a re-establishment of homologous pairing at the transition from mitosis to meiosis. Spermatogenesis completely dispenses with synapsis and recombination; cohesins and lateral elements of the SC are not present (Meyer, 1964; Meyer, 1969; Rasmussen, 1973), and there is a complete lack of crossing over (CO) (Morgan, 1914). Connections between homologous chromosomes, including sex chromosomes, are performed by a surrogate mechanism based on a protein complex consisting of at least two proteins: Stromalin in Meiosis (Snm) and Mod (Mdg4) in Meiosis (MNM) (Thomas et al., 2005; reviewed by McKee et al., 2012). Moreover, sex chromosome pairing is governed by the presence of nucleolar genes (reviewed in McKee, 2009; Tsai and McKee, 2011; McKee et al., 2012), so it has been suggested that rDNA would have a similar function to the pairing centres (PCs) described below in *C. elegans* (Tsai and Mckee, 2011). In support of this idea, it has been observed that an insertion or deletion of rDNA affects sex chromosome pairing and, not only that but, only a few copies of intergenic spacer regions of rDNA are enough to promote pairing (McKee and Karpen, 1990; McKee et al., 1992; McKee, 1996).

### Caenorhabditis elegans

**FIGURE 4:**
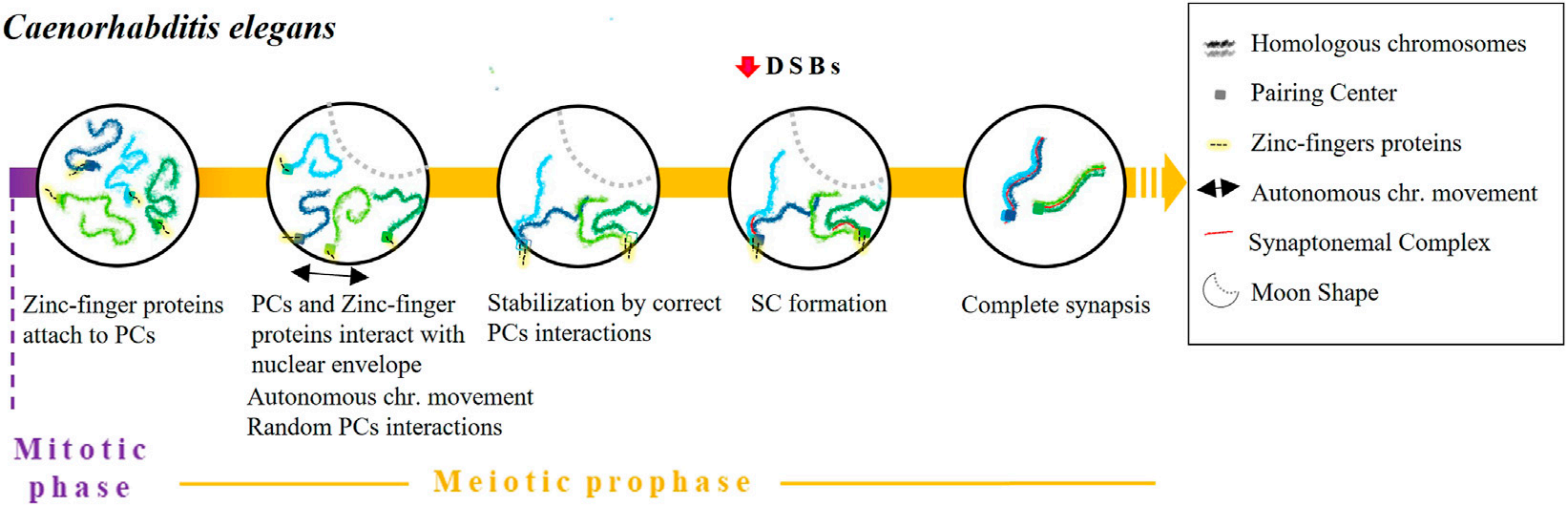
Timing and mechanisms of DSB-independent homologous pairing in *Caenorhabditis elegans*.

The pairing process of *C. elegans* begins at the onset of meiosis by a process that is independent of both DSBs and recombination (Dernburg et al., 1998; McKim et al., 1998) (Figure 4). During the leptotene/zygotene stage, chromatin assumes a half-moon shape (Hirsh et al., 1976) in which the nucleolus locates at the edges (Mlynarczyk-Evans and Villeneuve, 2017). Each chromosome of *C. elegans* contains a single subtelomeric region characterised by repeated DNA sequences widely referred to as Pairing Centres (PC). PCs promote and stabilise pairing and synapsis and are indispensable for accurate homologous segregation (Albertson et al., 1997; MacQueen et al., 2005). Some pieces of evidence indicate that PCs themselves are enough for chromosomes to recognise each other. For instance, pairing and synapsis take place transiently or inefficiently between chromosomes lacking PCs (MacQueen et al., 2005). Moreover, in reciprocal translocation chromosomes that are partly homologous and partly heterologous, pairing always begins in the PC region which is shared by both chromosomes (MacQueen et al., 2005).

Various studies have detailed how PCs promote pairing. First, the alignment of homologous chromosomes is stabilised in a synapse-independent manner (MacQueen et al., 2002; 2005). Indeed, in the absence of synapsis (*syp-1 or syp-2* mutants) transient pairing occurs during the leptotene and zygotene stages (MacQueen et al., 2002; Colaiácovo et al., 2003). We know that a set of zinc-finger proteins encoded in a single gene cluster - HIM-8, ZIM-1, ZIM-2 and ZIM-3—recognise and attach to a specific 12 bp repeat region present in PCs (Phillips et al., 2009). After binding, the resulting complexes interact with SUN-1 to form a bridge that crosses the nuclear envelope in a similar way to how telomeres form a *bouquet* structure. This mechanism is considered a variant of the *bouquet* (Penkner et al., 2009; Sato et al., 2009) although, in this case, the PCs are never completely clustered (Wynne et al., 2012). Afterward, chromosomes move through the nuclear envelope to ease homologous recognition by causing random interactions of PCs until they stabilise with the corresponding homologous PC and the formation of the SC (Baudrimont et al., 2010; Mlynarczyk-Evans and Villeneuve, 2017). SC central element polymerisation typically begins in proximity to PCs, although SC formation can still occur even without the participation of PCs (MacQueen et al., 2005; Hayashi et al., 2010). Importantly, it has been proposed that homologous synapsis is not reliant on recombination, as it occurs normally even in a *C. elegans spo-11* null mutant (Dernburg et al., 1998). Some researchers have proposed that chromosomal dynamics can prevent weak associations between non-homologous chromosomes. This mechanism is thought to be particularly important in cases where there is no stabilisation by PCs (Baudrimont et al., 2010; Wynne et al., 2012). Finally, various proteins have been described as being involved in meiotic prophase chromosome movements, including the meiotic family of serine/threonine protein kinases Polo-like kinases PLK-1 and PLK-2 (Lake et al., 2011), the motor protein dynein, the transmembrane SUN/KASH proteins and the orthologue of mammalian vinculin, DEB-1 (Rohožková et al., 2019). Interestingly, missense mutations in *sun-1* cause pairing defects and non-homologous synapsis (Penkner et al., 2007; Labrador et al., 2013). Moreover, homolog pairing is markedly delayed by dynein knockdown (Sato et al., 2009).

### Mus musculus

Some studies have shown that the association of homologous chromosomes in mouse germ cells takes place before the onset of meiosis (Boateng et al., 2013; Solé et al., 2022) or directly at the early leptotene stage (Ishiguro et al., 2014; Scherthan et al., 2014), in both cases before the formation of DSBs. Solé et al. (2022) quantified this process and demonstrated that up to 73.83% of homologous chromosomes are already in contact at premeiotic stages, suggesting the ability of homologous chromosomes to find each other before meiosis.

Boateng et al. (2013) showed that early pairing of homologous chromosomes in mice depends on the presence of SPO11 but not on its catalytic activity. The independence of pairing from SPO11 activity was confirmed later by Ishiguro et al. (2014). They observed pairing of homologous chromosomes in spermatocytes from *spo11* knockout mice, although less frequently than in wild-type spermatocytes, particularly in the early zygotene stage. Ishiguro and others also postulated that cohesins would guide homologous pairing. This idea was based on two observations. First, during the first meiotic prophase, the distribution pattern of cohesins RAD21L and REC8 appeared to be unique along each chromosome but identical in each homolog (Ishiguro et al., 2011). Second, homologous chromosome pairing in mice *rad21l−/−* mutants was impaired, suggesting a relevant role for this cohesin in the DSB-independent early pairing. Conversely, homolog pairing was observed in a significant population of *rec8−/−* mice spermatocytes (Ishiguro et al., 2014). Supporting the participation of cohesins, Ding et al. (2016a), Ding et al. (2016b) also observed an alteration of the pairing pattern in *S. pombe* in the absence of Rec8 and Pds5.

**FIGURE 5:**
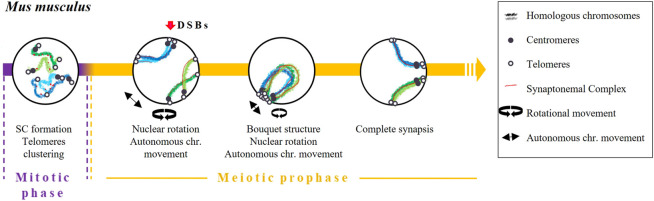
Timing and mechanisms of DSB-independent homologous pairing in *Mus musculus*.

The independent pairing of DSBs in mice also appears to be regulated by the expression of certain prophase I genes during spermatogonia proliferation, such as some components of SC and REC8 proteins (Wang et al., 2009; Elkouby et al., 2016) (Figure 5). Rubin et al. (2020) proposed that the expression of SC proteins prior to the onset of meiosis may resemble the expression of transverse filaments and central elements [C (3)G and Corona (CONA), respectively] in *D. melanogaster*. Indeed, Bisig et al. (2012) described an association of telomeres (although not specifically homologous telomeres) and, consequently, of centromeres in type B spermatogonia and pre-leptotene mice spermatocytes. Interestingly, this association was altered in the absence of SYCP3 (Bisig et al., 2012).

Early pairing of homologous chromosomes later became reinforced by the *bouquet* structure formation and chromosome dynamics. This structure facilitates the interaction of different chromosomal interstitial points. In terms of dynamics, a combination of two movements take place during prophase: nuclear rotation and an autonomous movement of the chromosomes (Conrad et al., 2008; Shibuya et al., 2014; Lee et al., 2015; Spindler et al., 2019). When the bouquet structure and chromosome dynamics are altered, a reduction in homologous pairing and synapsis has been observed (Shibuya et al., 2014). Finally, pairing will be completely stabilised through the repair mechanisms of DSBs (recombination) and the formation of the SC (Baudat et al., 2000).

## Final remarks

Table 1 summarises the main characteristics of early homologous pairing in the five model organisms reviewed in this work. The clustering of telomeres (or distal regions in the case of *C. elegans*) and/or centromeres appear to be a common mechanism in the early steps of the process. This chromosome disposition would place homologous chromosomes at the same latitude of the nucleus, orienting their chromosome arms and, therefore, helping the alignment of homologous regions for a more efficient homology search. The fact that the clustering occurs at a specific region of the nuclear envelope and before the initiation of chromosomal movements, would prevent the formation of “interlocks” between the chromatin of different chromosomes (images of these knots can be seen in Wang et al., 2009). It should be noted that the clustering of telomeres in the bouquet structure usually occurs near the microtubule organising centre (MTOC; known as the SPB in yeast and fungi, and as the centrosome in *C. elegans* and other metazoans). It suggests that the MTOC could have a role in the *bouquet* structure formation and in causing oscillatory movements (Sawin, 2005; Sato et al., 2009) that ultimately help to promote homologous recognition. Dynamics is another common trait that plays an important role in early homologous pairing. Movements such as nuclear rotation, horsetail movement or the displacement of telomeres through the nuclear envelope have been suggested to have two objectives. It would first help to find those specific elements that facilitate pairing (SC structure, other proteins, RNA and/or DNA) by establishing strong interactions in these regions followed by propagation of pairing along the chromosome, and second, movements would eliminate weak interactions between non-homologous chromosomes. In fact, if there are alterations of proteins involved in chromosomal movement, the frequency of synapsis between heterologous chromosomes increases (Penkner et al., 2009).

**TABLE 1:** Elements involved in early meiotic pairing in different species (Chr.) chromosome, (SC) synaptonemal complex, (PCs) pairing centers. *In *Saccharomyces cerevisiae*, there is a centromere coupling mechanism that involves the proximity of homologous and non-homologous centromeres.

	When does homologous pairing begin?	Does homologous pairing begin before DSBs formation?	Does homologous pairing occur in the absence of DSBs formation or recombination?	Do these elements promote homologous pairing?						
				Centromere clustering	Telomere clustering	Chr. dynamics	SC	DNA sequences	RNA sequences	Cohesin meiotic components
*Saccharomyces cerevisiae*	Prophase onset	No*	Yes	Yes	Yes	Yes	Yes	No data	No data	Yes
*Schizosaccharomyces pombe*	Prophase onset	Yes	Yes	Yes	Yes	Yes	No data	No data	Yes (non-coding RNA)	Yes
*Drosophila melanogaster*	Mitotic phase	Yes	Yes	Yes	No	Yes	Yes	Yes (rDNA, pairing sites)	No data	No data
*Caenorhabditis elegans*	Prophase onset	Yes	Yes	No (Holocentric chromosomes)	No	Yes	No	Yes (PCs)	No data	No data
*Mus musculus*	Before prophase	Yes	Yes	Yes	Yes	Yes	Yes	No data	No data	Yes

Based on the information presented in this review, it becomes evident that the processes of homologous chromosome pairing encompass additional mechanisms before the repair of double-strand breaks (DSBs). Independent DSB repair mechanisms would drive homologous chromosomes to approach, facilitating the search for homology after DSBs formation. In this way, early pairing would prevent the search for homologous sequences in non-homologous chromosomes and, consequently, the formation of unwanted interactions. At the same time, these mechanisms would facilitate the repair of DSBs using the intact homologous duplex as a template.

Overall, it is crucial to shift our understanding of the chromosomal pairing process from being solely driven by recombination to a process promoted by multiple factors that overlap in time. A more comprehensive understanding of the factors involved in homologous pairing and how they interact with one another is essential to understand the mechanisms that govern chromosome stability. Future research should aim to identify and characterise these factors.
